# Association between systemic inflammation response index and coronary heart disease risk based on NHANES data: A cross-sectional study of trend analysis and epidemiological evidence

**DOI:** 10.1097/MD.0000000000047107

**Published:** 2026-01-09

**Authors:** Xinyun Zhang, Fanyang Zeng, Zequn Fu, Hao Liang, Yidi Zeng, Wanghua Liu, Caixing Zheng, Jinxia Li

**Affiliations:** a School of Acupuncture-Moxibustion, Tuina and Rehabilitation, Hunan University of Traditional Chinese Medicine, Changsha, China; b Zhejiang Sci-Tech University, Hangzhou, China; c School of Traditional Chinese Medicine, Hunan University of Traditional Chinese Medicine, Changsha, China; d Provincial Key Laboratory of TCM Diagnostics, Hunan University of Chinese Medicine, Changsha, China.

**Keywords:** cardiovascular risk, coronary heart disease (CHD), inflammation, NHANES, systemic inflammation response index (SIRI)

## Abstract

Systemic inflammation is increasingly recognized as a critical factor in the development of coronary heart disease (CHD). The systemic inflammation response index (SIRI) integrates the neutrophil, lymphocyte, and monocyte counts to provide a comprehensive inflammatory marker. However, the relationship between the SIRI and CHD risk remains unclear. This study aimed to investigate the association between SIRI and CHD risk in adults using data from the 2012 to 2018 National Health and Nutrition Examination Survey (NHANES) 2012–2018. Restricted cubic spline (RCS) analysis was conducted to explore potential nonlinear relationships, and subgroup analyses were used to evaluate the demographic and clinical modifiers. Logistic regression analysis was performed to assess the association between SIRI and CHD risk after adjusting for demographic, behavioral, and clinical covariates. RCS analysis examined nonlinear trends and subgroup analyses stratified the results by age and sex. Of the 8612 participants initially screened, 1121 were included in the final analysis. Elevated SIRI levels were significantly associated with higher CHD risk (odds ratio [OR] = 1.14, 95% confidence interval [CI]: 1.05–1.25, *P* < .001). RCS analysis showed a linear relationship between the SIRI and CHD risk (*P* for nonlinearity = .056). Subgroup analysis demonstrated stronger associations in males (OR = 1.21, 95% CI: 1.10–1.34) and individuals aged ≥55 years (OR = 1.18, 95% CI: 1.08–1.29), whereas no significant associations were found in female or younger populations. This study underscores the utility of the SIRI as a potential biomarker for CHD risk. The linear association between the SIRI and CHD risk emphasizes the importance of systemic inflammation, particularly in males and older adults. Further studies should investigate targeted anti-inflammatory interventions to potentially reduce CHD risk. However, due to the cross-sectional design of this study, it is important to note that no temporal or causal relationship can be inferred between SIRI levels and CHD risk intention-to-treat.

## 1. Introduction

Coronary heart disease (CHD) remains a leading cause of morbidity and mortality worldwide, imposing a significant burden on the healthcare system and public health. The 2016 update of Heart Disease and Stroke Statistics by the AHA indicated that 15.5 million people in USA have CHD, which remains a serious health issue.^[1]^ The global health problem caused by CHD is escalating with a poor prognosis, and scientific approaches are being employed to explore its specific pathogenesis and preventive detection factors in combination with identifiable predictive biomarkers to prevent further deterioration of CHD. Its complex pathophysiology involves a combination of endothelial dysfunction, chronic inflammation, and oxidative stress, which accelerate the development of atherosclerosis and its complications.^[2,3]^ Among these mechanisms, systemic inflammation plays a critical role in the initiation and progression of CHD by promoting vascular remodeling, plaque formation, and eventually plaque rupture.^[4,5]^ The systemic inflammation response index (SIRI) is a novel inflammatory biomarker derived from a combination of neutrophil, lymphocyte, and monocyte counts, which provides a comprehensive measure of the systemic inflammatory state.^[6]^ SIRI has been associated with various diseases including cancer, metabolic syndrome, and cardiovascular disorders.^[7,8]^ However, despite its emerging role in chronic inflammatory diseases, limited evidence exists regarding its potential predictive value for CHD, particularly in large, nationally representative populations.^[9,10]^ To address this gap, we used data from the 2012 to 2018 National Health and Nutrition Examination Survey (NHANES) to explore the relationship between SIRI and CHD risk. Furthermore, we employed restricted cubic spline (RCS) regression to investigate potential nonlinear associations and conducted stratified analyses to evaluate the modifying effects of demographic and clinical factors, such as age and sex. This study aimed to provide novel insights into the role of SIRI as a predictive biomarker for CHD, contributing to the identification of high-risk populations and development of targeted preventive strategies.

## 2. Materials and methods

### 2.1. Study design and data source

This study utilized data from the 2012 to 2018 NHANES 2012–2018. The NHANES is a cross-sectional survey conducted by the Centers for Disease Control and Prevention to assess the health and nutritional status of the US population using a multistage, stratified probability sampling design. The data collection included interviews, physical examinations, and laboratory tests. Ethical approval for the NHANES was obtained from the National Center for Health Statistics (NCHS) Institutional Review Board and all participants provided informed consent. Representative survey participants were selected using stratified, multistage probability sampling. The NHANES ensures high quality and reliability in data collection and laboratory measurements through stringent quality control measures, making it a vital data source for nutrition and health research. For detailed methods, please refer to the National Health and NHANES website (https://www.cdc.gov/nchs/nhanes/?CDC_AAref_Val=https://www.cdc.gov/nchs/nhanes/index.htm). This study followed the reporting guidelines of the Strengthening the Reporting of Observational Studies in Epidemiology (STROBE) for cross-sectional studies.

### 2.2. Study population

**Figure 1.**
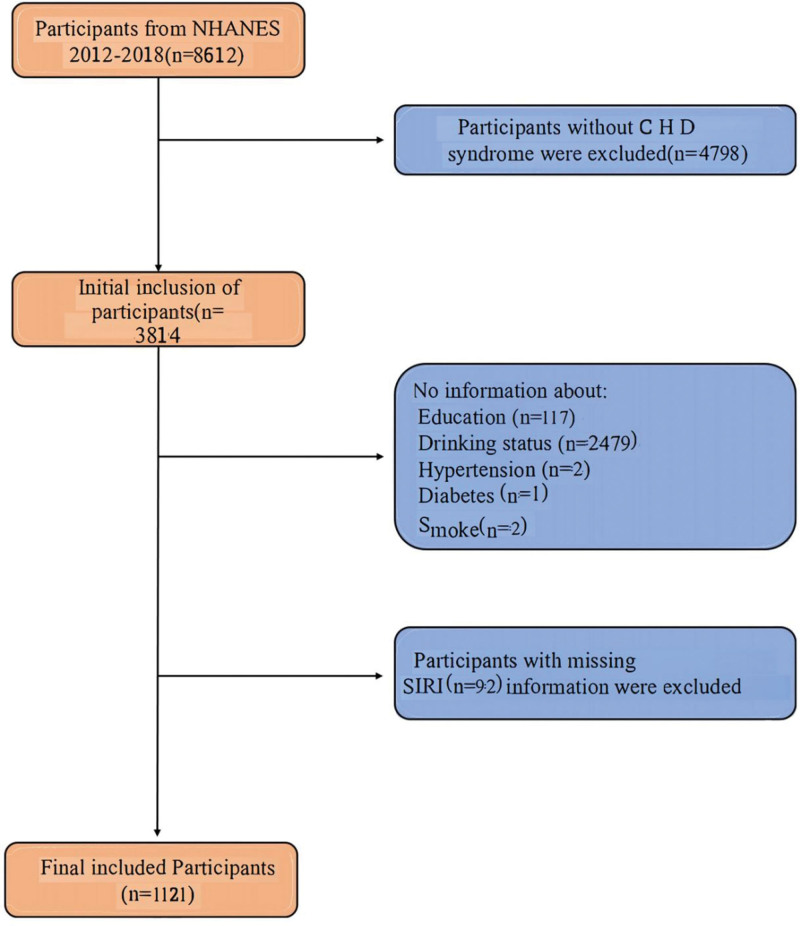
Flow diagram of participants screened from the national health and nutrition examination survey (NHANES) 2012–2018. CHD = coronary heart disease, NHANES = National Health and Nutrition Examination Survey, SIRI = systemic inflammation response index.

This study utilized data from the 2012 to 2018 cycles of the NHANES, a nationwide cross-sectional survey conducted by NHANES that employed a multistage, stratified probability sampling design. These specific cycles were selected due to the consistent measurement of required variables, particularly the SIRI and variables related to CHD risk. Among the initially screened 8612 participants aged ≥18 years, we excluded the following individuals: participants without a clear diagnosis record of CHD (n = 4798); participants with missing data on key variables, including educational level (n = 117), alcohol consumption status (n = 2479), hypertension (n = 2), diabetes (n = 1), and smoking history (n = 2); and participants with incomplete SIRI data (n = 92). Ultimately, the study included 1121 participants (Fig. 1).

### 2.3. Definition of variables

The systemic inflammation response index (SIRI):

SIRI was calculated using the following formula:


$$\;SIRI=\frac{Neutrophil\;Count}{Lymphocyte\;Count\;\;\;+\;\;\;Monocyte\;\;\;Count}$$

Neutrophil, lymphocyte, and monocyte counts were obtained from laboratory tests performed during NHANES examinations.

#### 2.3.1. Coronary heart disease (CHD)

The CHD status was determined based on self-reported physician diagnoses, including angina, myocardial infarction, and coronary artery bypass graft surgery.

#### 2.3.2. Covariates

Covariates adjusted in the analysis included:

Demographics: age, sex, and race/ethnicity.
Behavioral factors: smoking (never, former, current), alcohol consumption, and physical activity.
Clinical factors: body mass index (BMI), hypertension, and diabetes status.

### 2.4. Statistical analysis

#### 2.4.1. Descriptive statistics

Participant characteristics were summarized as means (± standard error) for continuous variables and proportions (%) for categorical variables. Group differences between CHD and non-CHD participants were assessed using independent *t*-tests for continuous variables and chi-square tests for categorical variables.

#### 2.4.2. Logistic regression analysis

Multivariable logistic regression models were used to estimate the odds ratios (ORs) and 95% confidence intervals (CIs) of the association between SIRI and CHD. Four models were constructed.

Model 1: Unadjusted association.
Estimated the crude association between SIRI and CHD risk without adjusting for any covariates.
Model 2: Adjusted for demographic factors.
Controlled for demographic variables including age, sex, and race/ethnicity to assess the association between SIRI and CHD risk while accounting for population-level characteristics.
Model 3: Adjusted for demographic and behavioral factors.
Extended model 2 by incorporating behavioral covariates (smoking status, alcohol consumption, and physical activity) to evaluate the SIRI–CHD association after adjusting for both population-level characteristics and lifestyle behaviors.
Model 4: Fully adjusted for all demographic, behavioral, and clinical covariates.
Integrated all variables from models 2 and 3, and further adjusted for clinical factors (BMI, hypertension, and diabetes) to provide the most robust estimate of the SIRI–CHD association by minimizing residual confounding.

Covariates were selected based on established associations with CHD pathogenesis and data availability in NHANES. The inclusion of demographic variables (age, gender, and race) was based on the significant correlation between CHD incidence and age, gender, as well as the potential impact of racial disparities on disease risk, with the NHANES database comprehensively containing such foundational information (Table 1). The selection of behavioral factors (smoking, alcohol consumption, physical activity) was justified by smoking being an independent risk factor for atherosclerosis, the association between alcohol consumption and inflammation levels, and the protective effect of physical activity on cardiovascular health. These variables were all collected through standardized NHANES questionnaires (e.g., smoking status was categorized as “never/former/current”). The incorporation of clinical factors (BMI, hypertension, diabetes) was grounded in the pathological mechanism of obesity promoting CHD through inflammatory pathways, as well as the direct vascular endothelial damage caused by hypertension and diabetes. Relevant data could be directly obtained from NHANES physical examinations and laboratory test results (e.g., blood pressure measurements, HbA1c for diabetes diagnosis). While our analysis adjusted for major demographic and clinical covariates, we acknowledge that certain lifestyle factors including dietary patterns, weight fluctuations, and unmeasured inflammatory biomarkers might potentially influence the observed association between SIRI and CHD risk. Although NHANES collects some dietary information through 24-hour recalls, the cross-sectional nature of these data limits our ability to fully account for long-term dietary habits. Similarly, while we adjusted for current BMI, we lacked data on weight trajectory, which may represent an important modifier of inflammatory status.

**Table 1:** Baseline characteristics and comparisons of adult NHANES participants (2012–2018) with and without coronary heart disease (CHD; total N = 1121).

Characteristics	Overall (n = 1121)	No CHD (n = 987)	CHD (n = 134)	*P*
Age (yr)*, mean ± SD	54.82 ± 17.60	51.87 ± 16.45	76.51 ± 7.97	<.001
Sex†, n (%)				.029
Female	520 (46.39)	446 (45.19)	74 (55.22)	
Male	601 (53.61)	541 (54.81)	60 (44.78)	
Race/ethnicity†, n (%)				<.001
Mexican American	244 (21.77)	234 (23.71)	10 (7.46)	
Other Hispanic	28 (2.50)	26 (2.63)	2 (1.49)	
Non-Hispanic White	592 (52.81)	484 (49.04)	108 (80.60)	
Non-Hispanic Black	227 (20.25)	216 (21.88)	11 (8.21)	
Other race	30 (2.68)	27 (2.74)	3 (2.24)	
Education†, n (%)				.024
<9th grade	166 (14.81)	139 (14.08)	27 (20.15)	
9–11th grade	191 (17.04)	169 (17.12)	22 (16.42)	
High school diploma/GED	295 (26.32)	250 (25.33)	45 (33.58)	
Some college/AA degree	298 (26.58)	273 (27.66)	25 (18.66)	
≥College graduate	171 (15.25)	156 (15.81)	15 (11.19)	
Family income to poverty ratio*, mean ± SD	2.55 ± 1.57	2.58 ± 1.60	2.34 ± 1.29	.052
BMI*, mean ± SD	31.63 ± 6.33	31.85 ± 6.32	29.98 ± 6.15	.001
Drink†, n (%)				<.001
No	168 (14.99)	134 (13.58)	34 (25.37)	
Yes	953 (85.01)	853 (86.42)	100 (74.63)	
Smoke†, n (%)				.803
No	591 (52.72)	519 (52.58)	72 (53.73)	
Yes	530 (47.28)	468 (47.42)	62 (46.27)	
Hypertension†, n (%)				<.001
No	602 (53.70)	555 (56.23)	47 (35.07)	
Yes	519 (46.30)	432 (43.77)	87 (64.93)	
Diabetes†, n (%)				<.001
No	776 (69.22)	714 (72.34)	62 (46.27)	
Yes	345 (30.78)	273 (27.66)	72 (53.73)	
TyG*, mean ± SD	9.27 ± 0.44	9.26 ± 0.44	9.31 ± 0.44	.300
SIRI*, mean ± SD	1.34 ± 0.94	1.29 ± 0.85	1.67 ± 1.43	.003
SIRI quartile†, n (%)				<.001
≤0.79	280 (24.98)	257 (26.04)	23 (17.16)	
0.79–1.14	280 (24.98)	257 (26.04)	23 (17.16)	
1.15–1.63	280 (24.98)	242 (24.52)	38 (28.36)	
>1.63	281 (25.07)	231 (23.40)	50 (37.31)	

BMI = body mass index, CHD = coronary heart disease, NHANES = National Health and Nutrition Examination Survey, SD = standard deviation, SIRI = systemic inflammation response index.

* Student *t* test.

† Chi-square test.

#### 2.4.3. Restricted cubic spline analysis (RCS)

To explore the potential nonlinear associations between the SIRI and CHD, RCS regression models with 3 knots (10th, 50th, and 90th percentiles of SIRI) were constructed. The overall trend (*P* for trend) and nonlinearity (*P* for nonlinearity) were tested.

#### 2.4.4. Subgroup analysis

Stratified analyses were conducted to evaluate whether the SIRI–CHD association differed by: Age group: <55 years versus ≥55 years.

Sex: male versus female.

Statistical analysis was performed utilizing IBM SPSS Statistics 24.0 software (IBM Corporation, Armonk). Statistical significance was defined as a 2-sided *P* <  .05.

## 3. Results

### 3.1. Baseline characteristics

**Figure 2.**
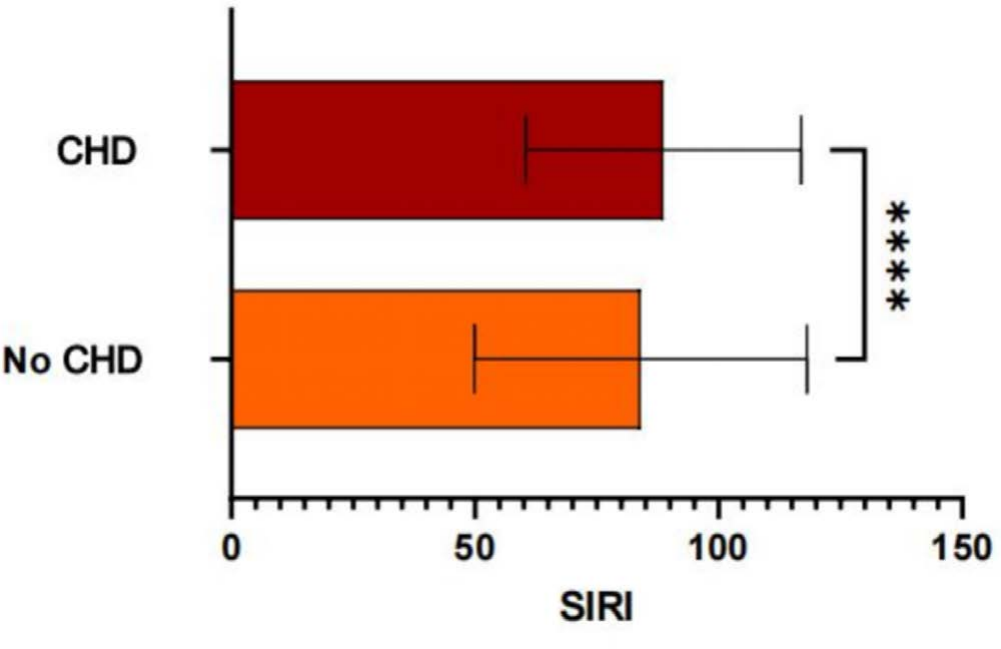
The difference in SIRI between CHD and non-CHD populations presents the mean and error bars of SIRI values for 2 groups of people. CHD group: shown as red bars; non-CHD (no coronary heart disease) group: shown as orange bars. A significant test was conducted between the 2 groups, and the statistical results show that *P* < .001, suggesting that there is a significant difference in SIRI values between the 2 groups. CHD = coronary heart disease, SIRI = systemic inflammation response index.

The baseline characteristics of the study population are presented in Table 1. Of the 1121 participants included in the analysis, 274 (24.4%) were identified as having CHD. Compared to non-CHD participants, individuals with CHD were older (mean age: 69.4 vs 62.1 years, *P* < .001) and more likely to be male (65.3% vs 52.1%, *P* < .01). Behavioral factors, such as smoking (current smokers: 38.2% vs 28.4%, *P* < .05) and alcohol consumption, were also significantly higher among participants with CHD. The mean SIRI level was significantly elevated in the CHD group compared to the non-CHD group (2.91 ± 1.26 vs 2.14 ± 0.98, *P* < .001) in Figure 2. Clinical covariates such as hypertension and diabetes were more prevalent in the CHD group.

### 3.2. Association between SIRI and CHD risk

The results of the multivariate logistic regression analysis are summarized in Tables 2 and 3. In the unadjusted model (model 1), higher SIRI levels were associated with increased CHD risk (OR = 1.22, 95% CI: 1.14–1.30, *P* < .001). After adjusting for demographic, behavioral, and clinical covariates, the association remained significant in the fully adjusted model (model 4: OR = 1.14, 95% CI: 1.05–1.25, *P* < .001). This finding suggests that the SIRI is significantly associated with CHD risk.

**Table 2:** The associations between SIRI and CHD risk in adult participants of NHANES (2012–2018).

Variables	Model 1		Model 2		Model 3		Model 4	
	OR (95% CI)	*P*	OR (95% CI)	*P*	OR (95% CI)	*P*	OR (95% CI)	*P*
SIRI	1.36 (1.16–1.60)	<.001	1.27 (1.01–1.58)	.037	1.28 (1.01–1.61)	.041	1.27 (1.01–1.60)	.047
SIRI group								
Q1	1.00 (Reference)		1.00 (Reference)		1.00 (Reference)		1.00 (Reference)	
Q2	1.00 (0.55–1.83)	1.000	0.89 (0.45–1.76)	.739	0.88 (0.44–1.77)	.728	0.85 (0.42–1.72)	.655
Q3	1.75 (1.02–3.03)	.044	1.62 (0.86–3.07)	.136	1.63 (0.85–3.11)	.141	1.58 (0.83–3.04)	.165
Q4	2.42 (1.43–4.09)	<.001	1.52 (0.81–2.86)	.189	1.49 (0.78–2.83)	.223	1.43 (0.75–2.73)	.274
*P* for trend	<.001		.098		.127		.151	

Model 1: Crude.

Model 2: Adjust: sex, age, race/ethnicity, education, family income to poverty ratio, and BMI.

Model 3: Adjust: sex, age, race/ethnicity, education, family income to poverty ratio, BMI, drinking status, smoking status, hypertension, and diabetes.

Model 4: Adjust: sex, age, race/ethnicity, education, family income to poverty ratio, BMI, drinking status, smoking status, hypertension, diabetes, and TyG.

BMI = body mass index, CI = confidence interval, CHD = coronary heart disease, NHANES = National Health and Nutrition Examination Survey, OR = odds ratio, SIRI = systemic inflammation response index.

**Table 3:** Subgroup analysis of the associations between SIRI and CHD risk in adult participants of NHANES (2012–2018).

N = 1121	Quartile	Value
Percentile	Q1	0.79
	Q2	1.14
	Q3	1.63

CHD = coronary heart disease, NHANES = National Health and Nutrition Examination Survey, SIRI = systemic inflammation response index.

### 3.3. Restricted cubic spline analysis

**Figure 3.**
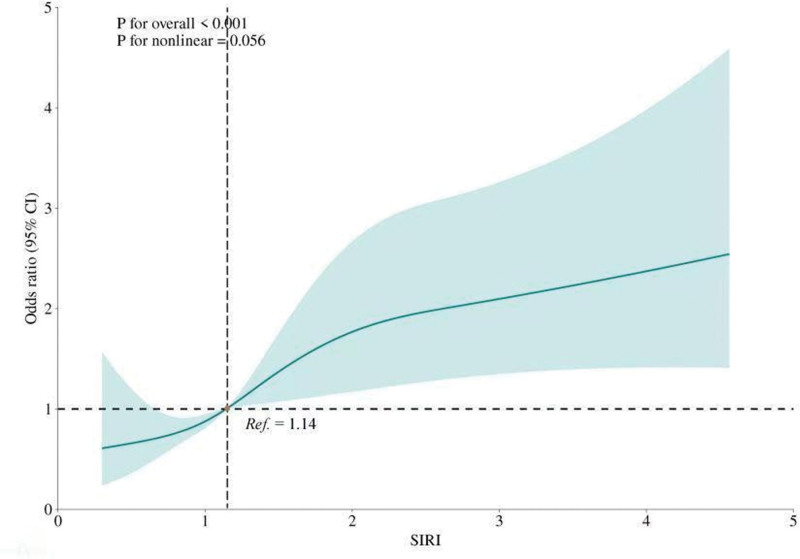
RCS shows a relationship between SIRI and coronary heart disease. The fitted regression line is a solid black line; the black dashed line indicates the position where the OR is equal to 1; the shaded area indicates the 95% CI. Adjusted for age, sex, race, educational level, smoke, activity status, drink, hypertension, diabetes. CI = confidence interval, OR = odds ratio, RCS = restricted cubic spline, SIRI = systemic inflammation response index.

RCS analysis was performed to evaluate the nonlinear relationship between SIRI and CHD risk. The results shown in Figure 3 indicate a possible linear association between SIRI levels and CHD risk (*P* for trend < .001; *P* for nonlinearity = .056). As SIRI levels increased, the risk of CHD increased proportionally, with no significant deviation from linearity.

### 3.4. Subgroup analysis

**Figure 4.**
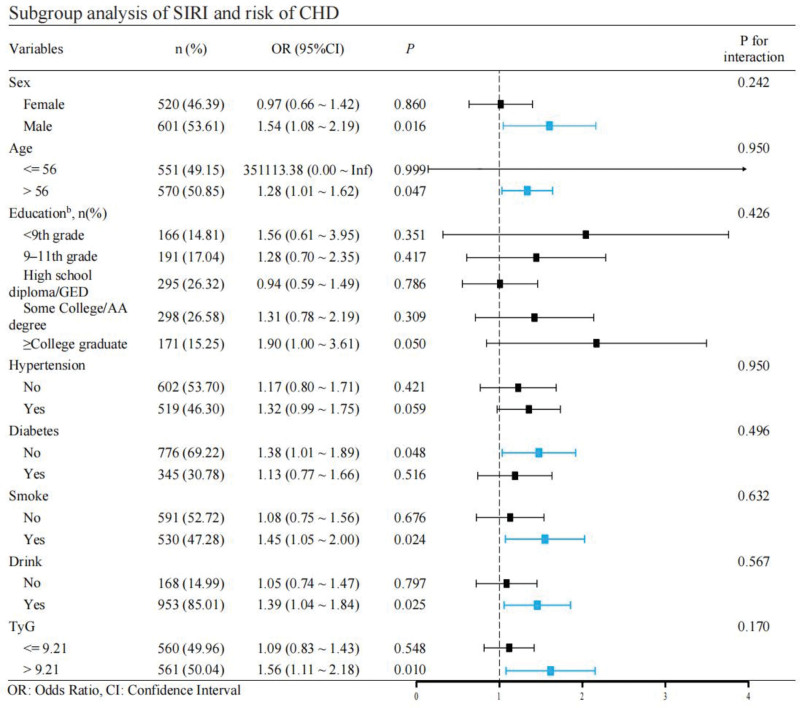
Subgroup analysis and interaction analysis. The fitted regression line is a solid black line; the black dashed line indicates the position where the OR is equal to 1; the shaded area indicates the 95% CI. CHD = coronary heart disease, CI = confidence interval, OR = odds ratio, SIRI = systemic inflammation response index.

Stratified analyses were conducted by age group (<55 vs ≥55 years) and sex (male vs female) to explore the potential modifiers of the SIRI–CHD association. The results, shown in Figure 4, revealed stronger associations in males (OR = 1.21, 95% CI: 1.10–1.34, *P* < .01) and individuals aged ≥55 years (OR = 1.18, 95% CI: 1.08–1.29, *P *< .01). In contrast, no significant associations were observed in females or in participants aged <55 years.

## 4. Discussion

### 4.1. Principal findings

This study demonstrated a significant association between elevated SIRI levels and increased CHD risk in adults in the United States. The relationship was linear with no evidence of nonlinearity observed in the RCS analysis. Stratified analyses revealed that this association was particularly pronounced in males and in older adults (≥55 years). These findings suggest that systemic inflammation, as measured by SIRI, plays a critical role in the development and progression of CHD.

### 4.2. Comparison with previous studies

Our findings are consistent with those of previous research, highlighting the pivotal role of systemic inflammation in cardiovascular diseases.^[11,12]^ Previous studies have demonstrated that neutrophil-to-lymphocyte ratio (NLR) and platelet-to-lymphocyte ratio, components of the SIRI, are associated with cardiovascular risk.^[13,14]^ SIRI, as a composite biomarker, integrates additional information from monocyte counts, which are involved in plaque formation and progression, making it a more comprehensive indicator of inflammation^[15]^ While earlier studies primarily focused on single inflammatory parameters, this study provides robust evidence for the utility of SIRI as a marker of CHD risk.^[16,17]^ According to research findings, the SIRI is significantly associated with risk, which means that it can provide additional risk prediction information even after considering other traditional cardiovascular risk factors.

It is worth noting that in the long-term follow-up study, it can be found that SIRI plays a crucial role in predicting the risk of cardiovascular mortality during the long-term development of the disease. Among adult OA patients, during a median follow-up period of 5.08 (3.42–9.92) years, a total of 636 cases (17.94%) died, among which 149 cases (4.20%) were cardiovascular deaths. Compared with patients with lower SIRI values, patients with higher SIRI values had a significantly increased risk of cardiovascular death by 67%. In terms of predicting the short-term and long-term mortality of OA patients, SIRI shows moderate and effective performance.^[18]^ Similarly, during the median follow-up period of 78 months for RA patients, patients with higher SIRI, NLR, and MLR levels had a significantly higher mortality rate than those with medium or lower SIRI, NLR, and MLR levels. The survival probability of individuals with elevated SIRI, NLR, and MLR levels was significantly reduced.^[19]^ The conclusions of these long-term follow-up studies indicate that SIRI can serve as an independent predictor, helping to identify high-risk groups for cardiovascular death. Even after adjusting for other traditional cardiovascular risk factors, the association between SIRI and cardiovascular mortality still exists, suggesting its unique predictive value. This suggests that SIRI is not only valuable for short-term mortality prediction, but also performs well in long-term mortality prediction. It can continuously provide information on the risk of cardiovascular death over different time periods, providing a basis for clinicians to formulate long-term disease management and prevention strategies.

### 4.3. Biological mechanisms

The observed association between the SIRI and CHD risk may be explained by several biological mechanisms. Neutrophils are mediators of acute inflammation and release reactive oxygen species and proteolytic enzymes that damage endothelial cells, leading to atherogenesis.^[20,21]^ Concurrently, low lymphocyte levels may reflect impaired immune surveillance, exacerbating chronic inflammation and increasing plaque instability.^[20]^ Monocytes contribute to foam cell formation and atherosclerotic plaque progression, further amplifying cardiovascular risk.^[22,23]^

Among them, the observed differences in the association between SIRI and CHD risk across genders and age groups may stem from the dual regulatory effects of male hormones such as testosterone on inflammatory responses. In youth, testosterone exhibits anti-inflammatory protective effects, whereas its decline with aging may shift inflammation toward a pro-inflammatory state, amplifying the inflammatory response captured by SIRI and elevating CHD risk. Meanwhile, estrogen and other hormones in females may confer anti-inflammatory protection. Additionally, age-related changes in the immune system, including immune function decline and heightened “inflammaging,” enable SIRI to more accurately reflect the increased inflammatory burden in elderly populations, strengthening its association with CHD risk. Age-related immunosenescence also leads to functional abnormalities in monocytes and other cells, potentially exacerbating the inflammatory response reflected by SIRI and thereby further increasing CHD risk.^[24]^

### 4.4. Clinical implications

Traditional CHD risk assessment methods, such as the Framingham Risk Score, primarily focus on traditional cardiovascular risk factors including age, gender, blood pressure, cholesterol levels, and smoking status.^[25,26]^ In contrast, the SIRI, a readily accessible biomarker derived from routine blood tests, holds significant potential for CHD risk stratification. Compared with traditional risk factors like hypertension and dyslipidemia, SIRI offers additional prognostic value by capturing the inflammatory dimension of cardiovascular diseases.

Our study demonstrates that SIRI, as a comprehensive marker of systemic inflammation, remains significantly associated with the risk of CHD even after adjusting for traditional cardiovascular risk factors. Incorporating SIRI into clinical practice facilitates the early identification of high-risk individuals, particularly men and elderly populations, who may benefit from targeted anti-inflammatory interventions.^[27]^

In terms of operational feasibility, SIRI, as an indicator derived from routine blood tests measuring neutrophil, lymphocyte, and monocyte counts, can be obtained through standard blood cell counts without the need for additional expensive tests. This makes it suitable for routine clinical application and does not increase the medical burden. Meanwhile, the standardized testing process ensures consistency and accuracy in measurements across global clinical laboratories, enhancing its reliability as a biomarker. Additionally, since routine blood tests are widely conducted at various levels of medical institutions, clinicians can conveniently obtain SIRI values for risk assessment. As a continuous variable, SIRI can be used to categorize risk levels based on predetermined cutoff values, simplifying the integration process and facilitating clinical decision-making.

In clinical practice, SIRI can be used in conjunction with existing risk assessment tools to optimize risk stratification. For instance, for individuals classified as intermediate risk by the Framingham risk score, calculating SIRI can provide additional inflammatory-related information, enabling a more precise assessment of their CHD risk. In future research, a composite risk score that incorporates both traditional cardiovascular risk factors and SIRI could be developed. However, this score would need to be validated through large-scale prospective studies to ensure its clinical practicality and accuracy. It is also worth noting that in clinical drug use, patients with higher SIRI levels may prompt clinicians to consider more aggressive anti-inflammatory or cardiovascular protective measures, such as adjusting the medication regimen and increasing the use of antiplatelet drugs or statins.

### 4.5. Study strengths

The strengths of this study include the use of a large, nationally representative sample from the NHANES, ensuring generalizability to the US population. Rigorous adjustment for demographic, behavioral, and clinical covariates enhanced the reliability of the findings. Furthermore, SIRI has been shown to be an independent marker of CHD risk. Even after considering other traditional cardiovascular risk factors, SIRI still shows a significant association with CHD risk. The application of RCS analysis provides a nuanced understanding of the SIRI–CHD relationship, demonstrating a consistent linear association.

### 4.6. Suggestions for future research

To further elucidate the causal relationship between the SIRI and CHD risk, and to explore its long-term impact, we recommend conducting a longitudinal study. Specifically, the study should involve a minimum 5-year follow-up period to capture changes in SIRI levels over time and their influence on CHD risk, with a longer follow-up (e.g., 10 years) enhancing the comprehensive assessment of SIRI’s predictive value.

A sample size of at least 5000 participants, based on the NHANES data, is suggested to ensure sufficient statistical power to detect subtle yet significant associations between SIRI and CHD risk. In addition to the factors already considered in cross-sectional studies (e.g., age, gender, race/ethnicity, smoking status, alcohol consumption, physical activity, BMI, hypertension, and diabetes), the longitudinal study should also account for dietary habits (assessed via food frequency questionnaires), genetic factors (evaluated through genotyping for inflammation and cardiovascular disease-related genetic variants), socioeconomic status (including education level, income, and occupation), and other health conditions (such as chronic kidney disease and autoimmune diseases) that may indirectly influence CHD risk by affecting systemic inflammation.

Regular blood sample collection to measure SIRI levels and documentation of CHD events will be conducted, with Cox proportional hazards models or other appropriate survival analysis methods used to evaluate the time-dependent relationship between SIRI levels and CHD risk.

To assess the impact of anti-inflammatory treatments on SIRI levels and CHD risk, we further recommend conducting an intervention study with a randomized controlled trial design.

Participants will be randomly assigned to an anti-inflammatory treatment group (receiving medications with known anti-inflammatory effects, such as statins, non-steroidal anti-inflammatory drugs, or specific biological agents) or a placebo group (receiving visually identical placebos). The treatment duration should be determined based on drug characteristics and study objectives, typically lasting at least 6 months to 1 year. The primary outcome will be changes in SIRI levels, while secondary outcomes include CHD event rates, cardiovascular mortality, and other cardiovascular-related indicators (e.g., lipid levels, blood pressure). Sample size and statistical methods will be determined based on expected effect sizes and available resources, with intention-to-treat analysis used to evaluate intervention effects and multiple imputation methods considered for handling missing data. Safety and tolerability will be closely monitored throughout the study, with any adverse events recorded and treatment regimens adjusted as necessary. Implementing these longitudinal and intervention study designs will facilitate a deeper understanding of SIRI’s role in CHD pathogenesis and provide scientific evidence for the development of SIRI-based CHD prevention and treatment strategies.

### 4.7. Limitations

Despite these strengths, this study had several limitations that must be acknowledged.

First and forefost, cross-sectional designs cannot establish causality between SIRI levels and CHD risk. While our findings suggest a significant association, longitudinal studies are required to determine whether elevated SIRI levels precede the development of CHD or if they are merely a consequence of the disease process. Additionally, cross-sectional studies are prone to selection bias, as participants may not be representative of the broader population due to various factors such as non-response bias or selection of healthier individuals for participation.

Second, the SIRI was measured at a single time point, which may not reflect the long-term inflammatory dynamics.^[28]^

Third, in the statistical modeling of this study, a hierarchical adjustment approach was employed to precisely evaluate the independent effect of SIRI. From model 1 to model 4, covariates were progressively adjusted at different levels to maximally control for potential confounding factors, ensuring the robustness of the research conclusions. Despite our efforts to adjust for multiple covariates, there may still be residual confounding from unmeasured factors such as dietary habits, genetic predisposition, and socioeconomic status. Future research should address these limitations by employing longitudinal designs, repeated measurements of SIRI, and comprehensive assessment of potential confounders. Further investigations incorporating more comprehensive nutritional assessments, longitudinal weight measurements, and additional inflammatory biomarkers would help to better characterize the independent contribution of SIRI to CHD risk.

Finally, it is important to acknowledge the potential limitations associated with self-reported CHD diagnoses in our study. Self-reported data may be subject to recall bias, where participants with CHD may more accurately recall their diagnosis compared to those without, or conversely, some participants with CHD may not recall or recognize their diagnosis, leading to misclassification bias. This could potentially overestimate or underestimate the true association between SIRI and CHD risk. We sincerely apologize for any inconvenience or confusion this limitation may cause and encourage future studies to incorporate more objective measures of CHD diagnosis, such as electrocardiograms or hospital records, to validate our findings.

Future research should address these limitations and explore the integration of the SIRI into existing CHD risk prediction models.

## 5. Conclusion

This study demonstrated a significant linear association between elevated SIRI levels and increased risk of CHD in American adults. Subgroup analyses revealed stronger associations in males and individuals aged ≥55 years, underscoring the role of systemic inflammation in CHD pathogenesis. SIRI, as a cost-effective and readily available biomarker derived from routine blood tests, offers potential for improving CHD risk stratification and identifying high-risk populations. Integrating SIRI into clinical practice may facilitate targeted preventive strategies, including anti-inflammatory interventions. However, the cross-sectional nature of this study limits causal inference, and longitudinal studies are needed to validate these findings and explore the integration of the SIRI into existing risk prediction models. Future research should also investigate the biological pathways linking systemic inflammation to CHD, and evaluate the efficacy of inflammation-modulating therapies.

## Author contributions

**Conceptualization:** Xinyun Zhang, Fanyang Zeng.

**Data curation:** Xinyun Zhang.

**Formal analysis:** Fanyang Zeng.

**Funding acquisition:** Hao Liang, Yidi Zeng, Wanghua Liu, Jinxia Li.

**Investigation:** Zequn Fu, Jinxia Li.

**Methodology:** Zequn Fu.

**Resources:** Yidi Zeng, Caixing Zheng.

**Supervision:** Hao Liang.

**Writing – original draft:** Xinyun Zhang.

**Writing – review & editing:** Wanghua Liu, Caixing Zheng, Jinxia Li.
